# Oxidative balance score predicts chronic kidney disease risk in overweight adults: a NHANES-based machine learning study

**DOI:** 10.3389/fnut.2025.1641496

**Published:** 2025-07-16

**Authors:** Leying Zhao, Cong Zhao, Yuchen Fu, Xiaochang Wu, Xuezhe Wang, Yaoxian Wang, Huijuan Zheng

**Affiliations:** ^1^Dongzhimen Hospital, Beijing University of Chinese Medicine, Beijing, China; ^2^Beijing University of Chinese Medicine, Beijing, China; ^3^Renal Research Institution of Beijing University of Chinese Medicine, Beijing, China; ^4^Henan University of Chinese Medicine, Zhengzhou, China

**Keywords:** oxidative balance score, chronic kidney disease, machine learning, overweight, precision nutrition

## Abstract

**Background:**

Oxidative stress plays a pivotal role in the pathogenesis of chronic kidney disease (CKD), particularly in overweight and obese populations where adipose tissue dysfunction exacerbates systemic inflammation and metabolic derangements. The oxidative balance score (OBS) is a composite index that integrates dietary antioxidants and pro-oxidant exposures, offering a quantifiable surrogate of oxidative burden. However, its utility in CKD prediction among overweight adults remains unclear.

**Methods:**

We analyzed data from 28,377 overweight or obese participants in ten NHANES cycles (1999–2018). OBS was calculated based on 16 dietary components and 4 lifestyle factors. CKD was defined using KDIGO guidelines. Survey-weighted logistic regression models were used to assess the association between OBS and CKD, with multivariable adjustment. Restricted cubic spline regression examined dose–response patterns, and subgroup analyses evaluated effect modifiers. Additionally, 14 machine learning algorithms were trained and validated using SMOTE-balanced data and five-fold cross-validation. Model interpretability was enhanced through SHapley Additive exPlanations (SHAP) analysis.

**Results:**

A higher OBS was inversely associated with CKD risk (fully adjusted OR per unit increase, 0.975; 95% CI, 0.969–0.981; *p* < 0.0001), with a significant linear dose–response relationship. This protective association was attenuated in morbid obesity (BMI ≥ 40 kg/m^2^; *P*_interaction_ < 0.001), a finding driven by the abrogation of the dietary score’s effect, while the lifestyle score remained protective in this subgroup. Among 14 machine learning models, GLMBoost was the top performer, achieving an Area Under the Curve (AUC) of 0.833 on the independent test set. SHAP analysis identified age, LDL-C, and SBP as primary predictors, but also revealed the significant protective contributions of OBS components—most notably physical activity and magnesium—and showed that age critically modifies the effects of both clinical and lifestyle factors.

**Conclusion:**

Higher OBS was associated with lower CKD risk in overweight and obese adults. This may support the role of oxidative balance in kidney health and its potential for early prevention strategies.

## Introduction

Chronic kidney disease (CKD) has emerged as a significant global public health concern, distinguished by its insidious onset, slow yet progressive course, and modifiable trajectory through early preventive interventions. In recent decades, the rising prevalence of obesity and metabolic disorders has fundamentally reshaped the epidemiological profile of CKD, placing obesity-related kidney dysfunction at the center of clinical and public health discourse. Epidemiological studies estimate that obesity-associated CKD accounts for approximately 2.7% of the general population, with obesity implicated in 15–30% of all CKD cases. Overweight individuals are reported to have a fivefold higher risk of developing CKD compared to those with normal body weight (1). Notably, a subset of these patients presents with atypical renal pathologies—such as tubulointerstitial injury and reduced glomerular filtration rate—in the absence of conventional nephropathic etiologies. These observations suggest that obesity-related CKD may represent a distinct clinical and pathophysiological phenotype, characterized by marked etiological heterogeneity and mechanistic complexity (2, 3).

Within this context, the early identification of high-risk overweight individuals and the prediction of renal functional decline—particularly through modifiable lifestyle and nutritional factors—remain pressing challenges in nephrological epidemiology and preventive nutrition. Oxidative stress, a well-recognized pathogenic nexus linking obesity, chronic inflammation, and renal dysfunction, is believed to play a pivotal role in the pathogenesis of this emerging CKD subtype. In individuals with excess adiposity, pro-inflammatory activation of adipose tissue, mitochondrial dysfunction, and dysregulation of the NADPH oxidase (NOX) enzyme system collectively drive the overproduction of reactive oxygen species (ROS). These elevated ROS levels contribute to renal microvascular injury, interstitial fibrosis, and progressive deterioration of kidney function (4).

The oxidative balance score (OBS) is a composite index that integrates both dietary and behavioral exposures to quantify an individual’s oxidative–antioxidative status. Unlike conventional approaches that focus on isolated nutrients or behaviors, OBS encompasses a wide range of dietary antioxidants and health-related behaviors, thereby providing a more holistic assessment of oxidative stress burden. OBS has shown promising predictive value for a variety of chronic diseases (5–7). However, its utility in predicting the risk of CKD among overweight individuals remains insufficiently studied. Considering the mechanistic complexity of obesity-associated CKD and the inherent limitations of existing predictive biomarkers, further investigation into OBS as a potential early indicator of renal injury and a tool to inform preventive strategies is clearly warranted.

Accordingly, this study utilizes data from the National Health and Nutrition Examination Survey (NHANES)—a nationally representative cohort in the United States—to investigate the relationship between OBS and the risk of CKD in overweight adults. Additionally, machine learning models enhanced by SHapley Additive exPlanations (SHAP) are employed to identify key predictive features and assess their relative contributions to CKD risk. The primary objective of this study is to evaluate the utility of OBS as an integrative biomarker that reflects lifestyle patterns, nutritional exposures, and oxidative stress in the context of primary CKD prevention. By improving risk stratification and enabling precision nutritional interventions for individuals with elevated body mass index (BMI), the findings aim to establish both a theoretical basis and practical framework for the early identification and management of obesity-associated CKD.

## Methods

### Study population and data source

This study utilized data from NHANES, a nationally representative surveillance program jointly conducted by the Centers for Disease Control and Prevention (CDC) and the National Center for Health Statistics (NCHS). NHANES employs a multistage, stratified probability sampling design to assess the health and nutritional status of the non-institutionalized U. S. population. All survey protocols were approved by the NCHS Research Ethics Review Board, and written informed consent was obtained from all participants.

**Figure 1:**
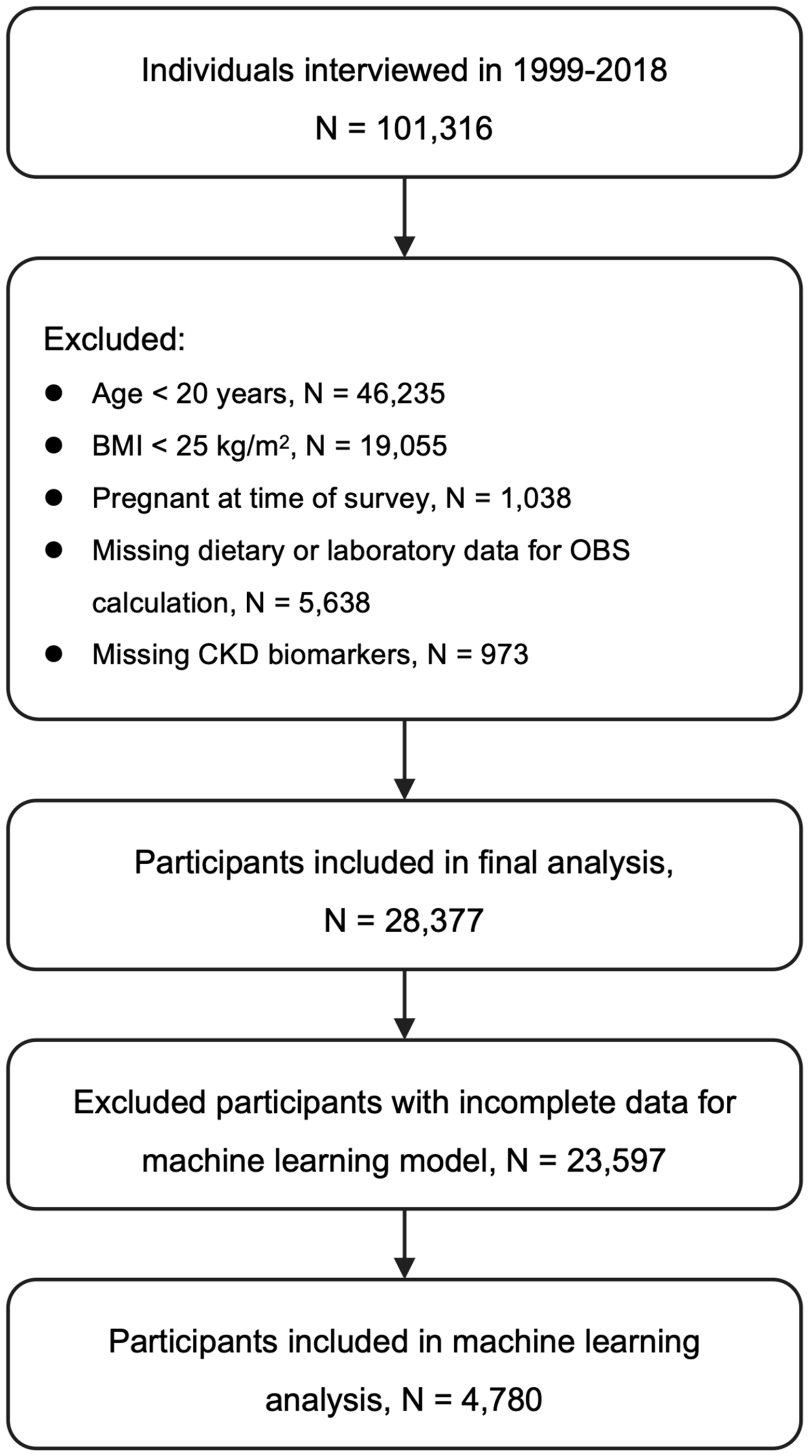
Flow of participant inclusion and exclusion. CKD, Chronic kidney disease; BMI, Body mass index; OBS, Oxidative balance score; eGFR, Estimated glomerular filtration rate.

Data from 10 consecutive NHANES cycles spanning 1999 to 2018 were pooled, yielding an initial sample of 101,316 individuals. To derive the final analytical cohort, the following exclusion criteria were sequentially applied: individuals aged <20 years (*n* = 46,235); those with a BMI < 25 kg/m^2^ (*n* = 19,055), in order to restrict the analysis to overweight and obese adults; pregnant women (*n* = 1,038); participants with missing key dietary or laboratory variables required to compute OBS (*n* = 5,638); and those lacking essential biomarkers for CKD evaluation, such as estimated glomerular filtration rate (eGFR) or urinary albumin data (*n* = 973). After applying these exclusions, a total of 28,377 overweight or obese adults were included in the final analysis (Figure 1).

### Definition of overweight

BMI was calculated as weight in kilograms divided by height in meters squared (kg/m^2^), with both measurements obtained by trained health technicians following standardized protocols at NHANES mobile examination centers (MECs). In accordance with the criteria established by the World Health Organization (WHO) and the National Institutes of Health (NIH), individuals with a BMI ≥ 25.0 kg/m^2^ were classified as overweight.

### Assessment of OBS

OBS is a composite metric designed to reflect an individual’s overall oxidative–antioxidative status. It was calculated based on 20 components, comprising 16 dietary nutrients—dietary fiber, carotenoids, riboflavin, niacin, vitamin B6, vitamin B12, vitamin C, vitamin E, total folate, calcium, magnesium, zinc, copper, selenium, total fat, and iron—and 4 lifestyle factors: physical activity, alcohol intake, serum cotinine, and BMI (8) (Supplementary Table S1). Dietary intake and alcohol consumption were assessed using two 24-h dietary recalls: the first was administered in person during the MEC visit, and the second was conducted via telephone 3–10 days later. Nutrient values were derived using the U. S. Department of Agriculture’s Food and Nutrient Database for Dietary Studies (FNDDS) for the 2017–2018 and 2019–2020 cycles. The average intake across both days was used to determine nutrient levels; when only one recall was available, that value was used. Physical activity was assessed via self-reported questionnaires. Metabolic equivalent task (MET) scores were assigned following NHANES protocols: vigorous-intensity work and leisure activities were given a score of 8.0, while moderate-intensity activities, walking, and bicycling for transportation were assigned scores of 4.0. Exposure to tobacco smoke was estimated using serum cotinine levels, measured by isotope-dilution high-performance liquid chromatography coupled with atmospheric pressure chemical ionization tandem mass spectrometry (ID HPLC-APCI MS/MS). BMI was calculated from directly measured weight and height. Each of the 20 OBS components was categorized into sex-specific tertiles. Antioxidant components (e.g., dietary fiber, vitamins, minerals, physical activity) were scored from 0 to 2 across the lowest to highest tertile. In contrast, pro-oxidant components (e.g., total fat, iron, serum cotinine, alcohol intake, and BMI) were scored in reverse order. The total OBS ranged from 0 to 40, with higher scores indicating a more favorable antioxidative profile. Participants were required to have valid data for at least 19 of the 20 OBS components to be included in the score calculation.

### Definition of CKD

The primary outcome of interest was the presence of CKD, defined according to the diagnostic criteria established by the Kidney Disease: Improving Global Outcomes (KDIGO) guidelines (9). eGFR was calculated using the updated Chronic Kidney Disease Epidemiology Collaboration (CKD-EPI) equation, incorporating serum creatinine, age, sex, and race. The albumin-to-creatinine ratio (ACR), expressed in mg/g, was derived from spot morning urine samples and served as an indicator of microalbuminuria. Participants were classified as having CKD if they met either of the following criteria: eGFR < 60 mL/min/1.73 m^2^ or ACR > 30 mg/g.

### Covariates

Covariates included demographic characteristics, clinical comorbidities, and prescription medication use obtained from NHANES. Demographic variables encompassed sex, age, race/ethnicity, marital status (married/living with partner vs. not), and educational attainment (below high school vs. high school and above). Clinical variables included hypertension, hyperlipidemia, diabetes mellitus, and atherosclerotic cardiovascular disease (ASCVD). Hypertension was defined as a mean systolic blood pressure ≥140 mmHg, diastolic blood pressure ≥90 mmHg, self-reported diagnosis, or current antihypertensive medication use. Hyperlipidemia was defined as triglycerides ≥150 mg/dL, total cholesterol ≥200 mg/dL, LDL-C ≥ 130 mg/dL, HDL-C ≤ 40 mg/dL in men or ≤50 mg/dL in women, or use of lipid-lowering therapy (10). Diabetes mellitus was identified by self-reported diagnosis or at least one of the following: HbA1c ≥ 6.5%, fasting glucose ≥7.0 mmol/L, random glucose ≥11.1 mmol/L, 2-h OGTT ≥11.1 mmol/L, or use of antidiabetic medications (11). ASCVD was based on self-reported physician diagnosis of coronary heart disease, angina, myocardial infarction, or stroke.

Prescription medication data were collected via in-home interviews using the NHANES drug questionnaire. Participants reported all prescription drugs taken in the previous 30 days, and interviewers verified information by inspecting medication containers (12). Drug names were matched to the Lexicon Plus® classification system (Cerner Multum, Inc.), updated annually to reflect U. S. market availability. Two nephrotoxic drug classes were evaluated: (1) renin–angiotensin system inhibitors (RASIs), including angiotensin-converting enzyme inhibitors (ACEIs) and angiotensin II receptor blockers (ARBs); and (2) nonsteroidal anti-inflammatory drugs (NSAIDs). Each was included as a binary variable (user vs. non-user) in multivariable models and subgroup analyses to account for potential confounding.

### Feature preprocessing for machine learning

A total of 36 candidate variables were initially selected for machine learning model development. To optimize model performance and ensure training stability, a multi-step preprocessing pipeline was implemented: Feature variance filtering: Variables with near-zero variance (≥95% identical values) were excluded from the analysis. Multicollinearity reduction: Pearson correlation coefficients were computed for continuous variables. For each pair with a correlation coefficient >0.8, one variable was removed to reduce redundancy. Normalization: All retained features were rescaled using MinMax normalization to mitigate the impact of differing numerical scales on model training. Class imbalance adjustment: The Synthetic Minority Oversampling Technique (SMOTE) was applied to the training set to address class imbalance and improve model generalizability. The final feature set encompassed demographic characteristics (e.g., sex, age, race/ethnicity, education level, marital status, and poverty-income ratio), biochemical markers (e.g., HbA1c, uric acid, C-reactive protein [CRP], TG, HDL-C, LDL-C, and blood pressure), and lifestyle/nutritional factors (e.g., alcohol intake, BMI, and intake of dietary vitamins and minerals).

### Machine learning model development and evaluation

Fourteen classification algorithms, encompassing both conventional and state-of-the-art machine learning techniques, were developed using the mlr3 framework. These included: CatBoost, support vector machine (SVM), random forests (implemented via rfsrc, ranger, and randomForest packages), XGBoost, LightGBM, gradient boosting machine (GBM), generalized linear model with elastic net regularization (GLMNet), GLMBoost, naïve Bayes, k-nearest neighbors (kNN), classification and regression tree (CART), and a feedforward neural network (nnet). All models were trained using default hyperparameters to enable fair and unbiased performance comparisons. The dataset was randomly partitioned into a training set (80%) and an independent test set (20%). Five-fold cross-validation was conducted within the training set to assess model stability and minimize performance variance. Model performance was primarily evaluated using the area under the receiver operating characteristic curve (AUC). After cross-validation, the model with the highest AUC was selected and further validated on the independent test set. Additional performance metrics—including accuracy, precision, recall, and F1-score—were also computed to comprehensively evaluate generalizability and classification robustness.

### Model interpretation using SHAP

To interpret model predictions and quantify the contribution of individual features, SHA*p* values were calculated for the best-performing model. SHAP is a state-of-the-art *post hoc* interpretability framework that estimates the marginal impact of each input variable on the model’s output, based on principles from cooperative game theory (13). This framework was employed to facilitate a multi-level interpretation of the model. Global feature importance was assessed by ranking predictors based on their mean absolute SHAP values. To move beyond simple feature ranking and specifically investigate complex, non-linear interactions, we generated SHAP dependence plots. These plots are designed to visualize how the marginal effect of a given feature on the model’s output is modified by the value of a second, interacting feature. This approach enhances model transparency and provides a deeper understanding of the synergistic effects between variables.

### Statistical analysis

All statistical analyses accounted for the complex, multistage sampling design of NHANES, incorporating sampling weights, stratification, and clustering. Continuous variables were summarized as means ± standard deviations (SD), while categorical variables were reported as frequencies and percentages. The association between OBS and CKD was evaluated using survey-weighted generalized linear models with a binomial distribution to fit logistic regression. Odds ratios (ORs) and their 95% confidence intervals (CIs) were reported, with covariates progressively adjusted across multiple models. Multivariable adjustment was conducted sequentially: Model 1 adjusted for race/ethnicity, age, sex, marital status, and educational attainment. Model 2 further adjusted for clinical comorbidities, including hypertension, hyperlipidemia, diabetes mellitus, and ASCVD. A final Model 3 was constructed by additionally adjusting for the use of NSAIDs and RASI. To assess potential non-linear dose–response relationships between OBS and CKD, restricted cubic spline (RCS) models were constructed using knots placed at the 10th, 50th, and 90th percentiles of OBS distribution. Subgroup and interaction analyses were performed across predefined categories, including age (20–59 vs. 60–80 years), sex, degree of obesity (BMI < 40 vs. ≥40 kg/m^2^), hypertension, hyperlipidemia, diabetes mellitus, ASCVD, and the use of RASI and NSAIDs, to explore potential effect modification.

Several sensitivity analyses were conducted to assess the robustness of our primary findings. First, to mitigate potential reverse causality where advanced CKD could influence participants’ diet and lifestyle, we repeated the primary analysis after excluding individuals with high-risk or very-high-risk CKD as defined by the KDIGO risk-prognosis heat map. The association between total OBS and CKD risk was then re-evaluated in the remaining cohort. Second, to address the equal weighting of the OBS construct and explore the contributions of its constituent parts, we deconstructed the total score into two distinct sub-scores: a lifestyle score (comprising the 4 components: physical activity, alcohol intake, serum cotinine, and BMI) and a dietary score (comprising the 16 nutritional components). The association between each of these continuous sub-scores and the odds of CKD was then assessed separately using the fully adjusted multivariable logistic regression model, and corresponding subgroup analyses were also performed. All analyses were conducted using R software (version 4.4.2). Key packages included survey, mlr3, mlr3benchmark, mlr3extralearner, kernelshap, and shapviz. A two-tailed *p*-value < 0.05 was considered statistically significant.

## Results

### Baseline characteristics

A total of 28,377 overweight and obese adults were included in the final analytical cohort. Participants were stratified according to the presence or absence of CKD, and their baseline characteristics are presented in Table 1. The overall mean age was 48.92 ± 0.19 years, with females accounting for 48.72% of the population. The average BMI was 31.75 ± 0.06 kg/m^2^, and the mean OBS was 19.84 ± 0.10. Compared to individuals without CKD, those with CKD were significantly older, more likely to be female, and had lower levels of educational attainment. The prevalence of former smoking and alcohol consumption was also significantly higher in the CKD group. Regarding clinical comorbidities, participants with CKD exhibited a markedly greater burden of hypertension, hyperlipidemia, ASCVD, and diabetes mellitus (*p* < 0.0001). In terms of metabolic parameters, individuals with CKD had significantly higher levels of HbA1c, TG, and SBP. Although HDL-C levels were slightly lower in the CKD group, the difference was not statistically significant (*p* = 0.73). Additionally, the mean BMI was significantly higher among participants with CKD (32.84 ± 0.14 vs. 31.57 ± 0.072; *p* < 0.0001), and their mean OBS was significantly lower compared to those without CKD (18.31 ± 0.14 vs. 20.10 ± 0.10; *p* < 0.0001).

**Table 1:** Baseline characteristics of participants.

	Total	Without CKD	With CKD	*p*-value
Age, years	48.92 ± 0.19	46.87 ± 0.19	60.88 ± 0.30	<0.0001
Sex, %				<0.0001
Male	14,227 (51.28)	11,613 (52.31)	2,614 (45.25)	
Female	14,150 (48.72)	11,316 (47.69)	2,834 (54.75)	
Ethnicity/race, %				<0.0001
White	12,680 (69.39)	10,192 (69.68)	2,488 (67.68)	
Black	6,137 (11.27)	4,673 (10.60)	1,464 (15.19)	
Mexican American	5,473 (8.70)	4,605 (8.93)	868 (7.36)	
Other races	4,087 (10.63)	3,459 (10.78)	628 (9.77)	
Education level, %				<0.0001
Below high school	7,608 (16.91)	5,761 (15.65)	1,847 (24.37)	
Above high school	20,748 (83.03)	17,154 (84.35)	3,594 (75.63)	
Marital status, %				<0.0001
Married or living with a partner	17,571 (66.00)	14,556 (67.97)	3,015 (59.39)	
Not married nor living with a partner	10,518 (32.93)	8,133 (32.03)	2,385 (40.61)	
Smoker, %				<0.0001
Now	17,775 (68.47)	15,004 (74.44)	2,771 (60.97)	
Former	5,102 (15.42)	3,723 (15.11)	1,379 (23.54)	
Never	3,704 (10.56)	2,821 (10.45)	883 (15.49)	
Alcohol user, %				<0.0001
Now	17,775 (68.47)	15,004 (74.44)	2,771 (60.97)	
Former	5,102 (15.42)	3,723 (15.11)	1,379 (23.54)	
Never	3,704 (10.56)	2,821 (10.45)	883 (15.49)	
Systolic blood pressure, mmHg	124.07 ± 0.18	122.44 ± 0.19	133.74 ± 0.38	<0.0001
Diastolic blood pressure, mmHg	72.22 ± 0.17	72.44 ± 0.18	70.89 ± 0.28	<0.0001
HbA1c (%)	5.68 ± 0.01	5.58 ± 0.01	6.26 ± 0.03	<0.0001
TG, mmol/L	1.66 ± 0.02	1.62 ± 0.02	1.91 ± 0.05	<0.0001
LDL, mmol/L	3.08 ± 0.01	3.10 ± 0.01	2.90 ± 0.02	<0.0001
HDL, mmol/L	1.29 ± 0.00	1.29 ± 0.00	1.28 ± 0.01	0.73
BMI, kg/m^2^	31.75 ± 0.06	31.57 ± 0.07	32.84 ± 0.14	<0.0001
OBS, score	19.84 ± 0.10	20.10 ± 0.10	18.31 ± 0.14	<0.0001
Comorbidities				
Hypertension, %	13,823 (43.85)	9,679 (39.08)	4,144 (71.75)	<0.0001
Hyperlipidemia,%	22,435 (78.87)	17,781 (77.67)	4,654 (85.91)	<0.0001
Diabetes mellitus,%	6,022 (16.26)	3,647 (12.41)	2,375 (38.76)	<0.0001
Atherosclerotic cardiovascular disease,%	3,235 (9.21)	1,838 (6.85)	1,397 (23.01)	<0.0001

### Association between OBS and CKD risk

In multivariable logistic regression analyses, a higher OBS was significantly associated with reduced odds of CKD (Table 2). When modeled as a continuous variable, each one-unit increase in OBS was linked to a 3.6% decrease in the odds of CKD in the unadjusted model (OR = 0.964; 95% CI: 0.959–0.969). This inverse association remained statistically significant, though slightly attenuated, after adjusting for demographic variables (Model 1: OR = 0.971; 95% CI: 0.965–0.977) and further for clinical comorbidities (Model 2: OR = 0.975; 95% CI: 0.969–0.982). Additional adjustment for the use of RASI and NSAIDs in the fully adjusted model (Model 3) did not materially change the association (OR = 0.975; 95% CI: 0.969–0.981). When OBS was examined in quartiles, a clear dose–response relationship emerged. In the fully adjusted model (Model 3), individuals in the highest OBS quartile (Q4) had 37.3% lower odds of CKD compared to those in the lowest quartile (Q1) (OR = 0.627; 95% CI: 0.548–0.717). Statistically significant and graded reductions in CKD risk were also observed in the second and third quartiles (Q2 and Q3). The test for linear trend across quartiles remained highly significant after full adjustment (*P*_trend_ < 0.0001).

**Table 2:** Multivariable-adjusted associations between OBS and CKD.

Groups	Crude model		Model 1		Model 2		Model 3	
	HR (95% CI)	*p*-value	HR (95% CI)	*p*-value	HR (95% CI)	*p*-value	HR (95% CI)	*p*-value
Continuous	0.964 (0.959, 0.969)	<0.0001	0.971 (0.965, 0.977)	<0.0001	0.975 (0.969, 0.982)	<0.0001	0.975 (0.969, 0.981)	<0.0001
Q1	ref	ref	ref	ref	ref	ref	ref	ref
Q2	0.780 (0.705, 0.863)	<0.0001	0.793 (0.711, 0.886)	<0.0001	0.803 (0.714, 0.904)	<0.001	0.797 (0.708, 0.897)	<0.001
Q3	0.626 (0.570, 0.688)	<0.0001	0.679 (0.613, 0.752)	<0.0001	0.707 (0.635, 0.788)	<0.0001	0.701 (0.629, 0.781)	<0.0001
Q4	0.509 (0.452, 0.574)	<0.0001	0.588 (0.516, 0.669)	<0.0001	0.633 (0.553, 0.724)	<0.0001	0.627 (0.548, 0.717)	<0.0001
P for trend		<0.0001		<0.0001		<0.0001		<0.0001

Low exposure (Q1) was used as the reference group. Model 1: ethnicity, age, sex, education, marital status.

Model 2: ethnicity, age, sex, education, marital status, hypertension, hyperlipidemia, ASCVD, DM.

Model 3: ethnicity, age, sex, education, marital status, hypertension, hyperlipidemia, ASCVD, DM, RASI, NSAID.

### Nonlinear relationship between OBS and CKD risk

**Figure 2:**
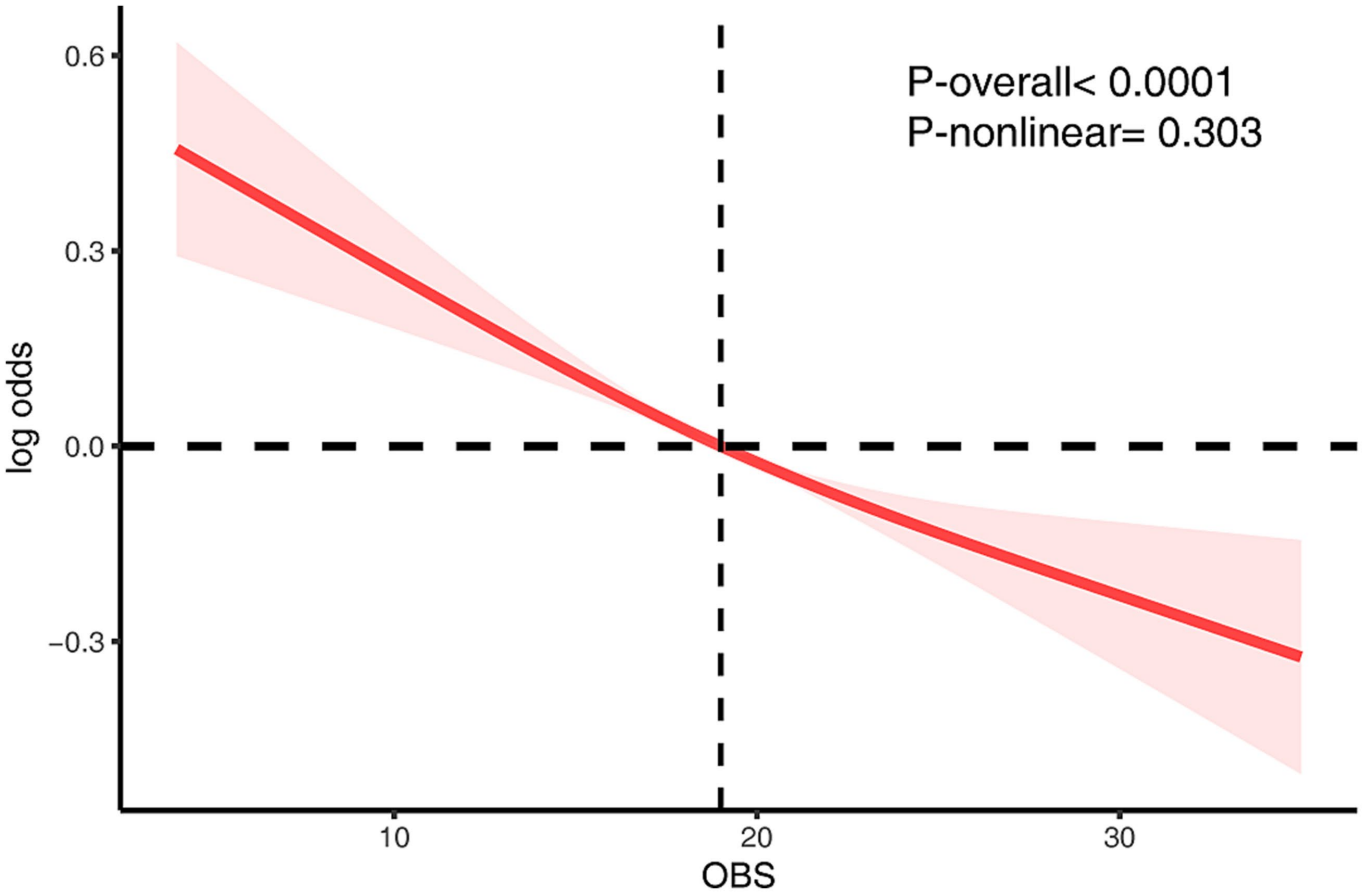
The RCS analysis of the association between OBS and CKD risk. The model was adjusted for age, sex, race/ethnicity, marital status, education, hypertension, hyperlipidemia, diabetes mellitus, ASCVD, RASI, and NSAID.

To further characterize the functional relationship between OBS and CKD risk, a RCS model was constructed (Figure 2). After full adjustment for covariates, the spline curve revealed a monotonic inverse association between OBS and CKD risk. Statistical analysis confirmed the overall significance of this relationship (*P*_overall_ < 0.0001), while the test for non-linearity was not statistically significant (*P*_non-linearity_ = 0.303), indicating a predominantly linear dose–response pattern.

### Subgroup analyses

**Figure 3:**
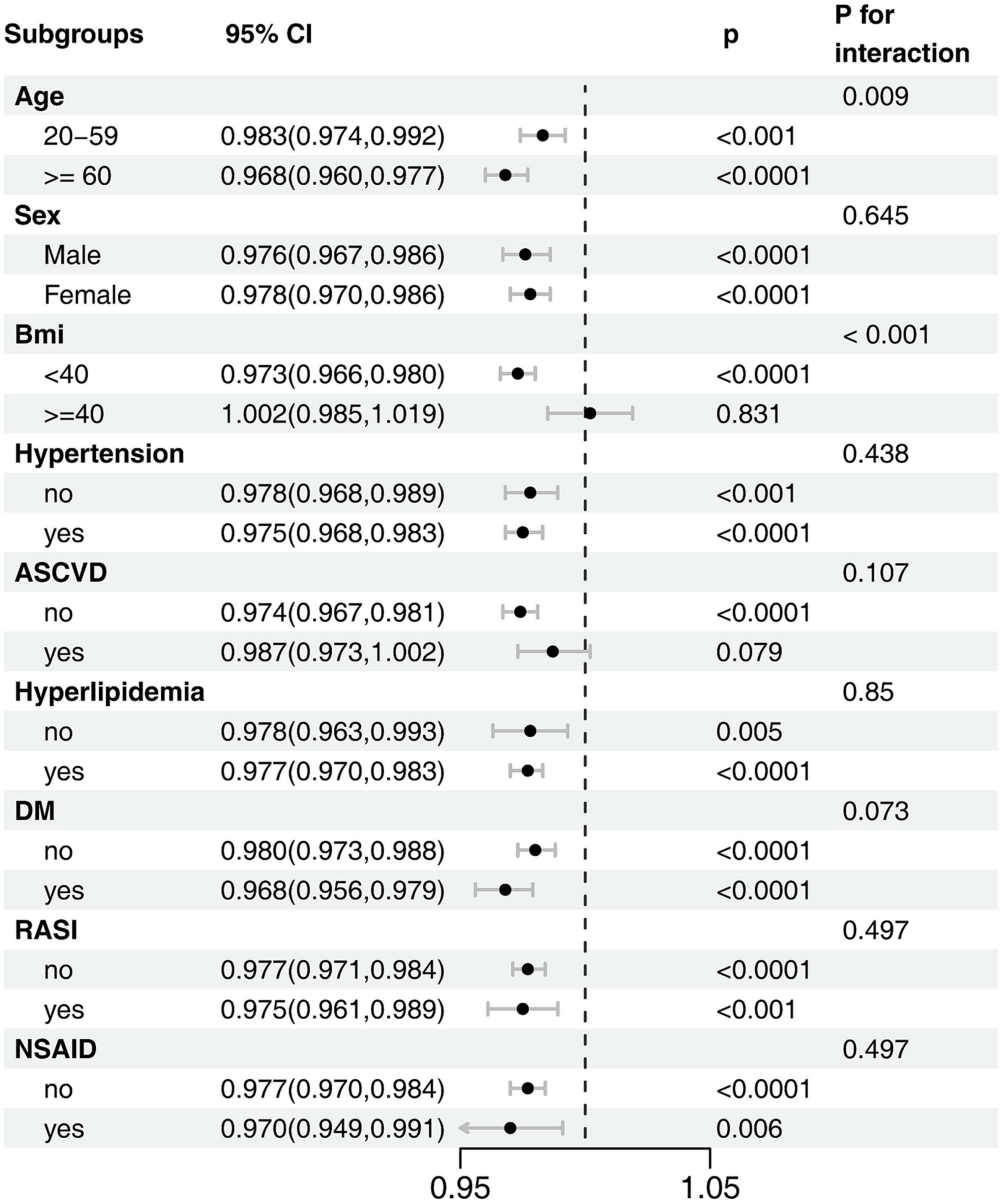
Subgroup analysis of the association between OBS and CKD risk. The model was adjusted for age, sex, race/ethnicity, marital status, education, hypertension, hyperlipidemia, diabetes mellitus, ASCVD, RASI, and NSAID.

To evaluate the consistency of this association, we performed subgroup analyses across several key demographic and clinical strata (Figure 3). The protective effect of a higher OBS was largely consistent across the majority of subgroups examined. However, we found statistically significant evidence of effect modification by both age (*P*_interaction_ = 0.009) and obesity status (*P*_interaction_ < 0.001). Notably, the interaction by obesity status revealed a critical threshold. While OBS was protective in individuals with a BMI < 40 kg/m^2^, this effect was completely abrogated in those with morbid obesity (BMI ≥ 40 kg/m^2^), for whom a higher OBS was no longer associated with lower odds of CKD (OR, 1.002; 95% CI, 0.985–1.019). The protective association was, however, more pronounced in older adults. No other significant interactions were detected, including for subgroups defined by comorbidities or medication use.

### Sensitivity analysis

To assess the robustness of our findings, we conducted two prespecified sensitivity analyses. The first was designed to mitigate potential reverse causality by repeating the primary analysis after excluding participants with high-or very-high-risk CKD. In this sub-cohort, the inverse association between a higher total OBS and CKD risk remained statistically significant after full multivariable adjustment (Model 3; OR, 0.980; 95% CI, 0.972–0.987) (Supplementary Table S2). The dose–response relationship was also consistent; individuals in the highest OBS quartile had 31.1% lower odds of CKD compared to those in the lowest quartile (Model 3 OR for Q4 vs. Q1, 0.689; 95% CI, 0.592–0.802) (Supplementary Table S3), and the test for linear trend remained significant (*P*_non-linearity_ < 0.0001) (Supplementary Figure S1). Further subgroup analysis of this cohort confirmed that the significant effect modification by morbid obesity persisted (*P*_interaction_ = 0.001) (Supplementary Figure S2).

**Figure 4:**
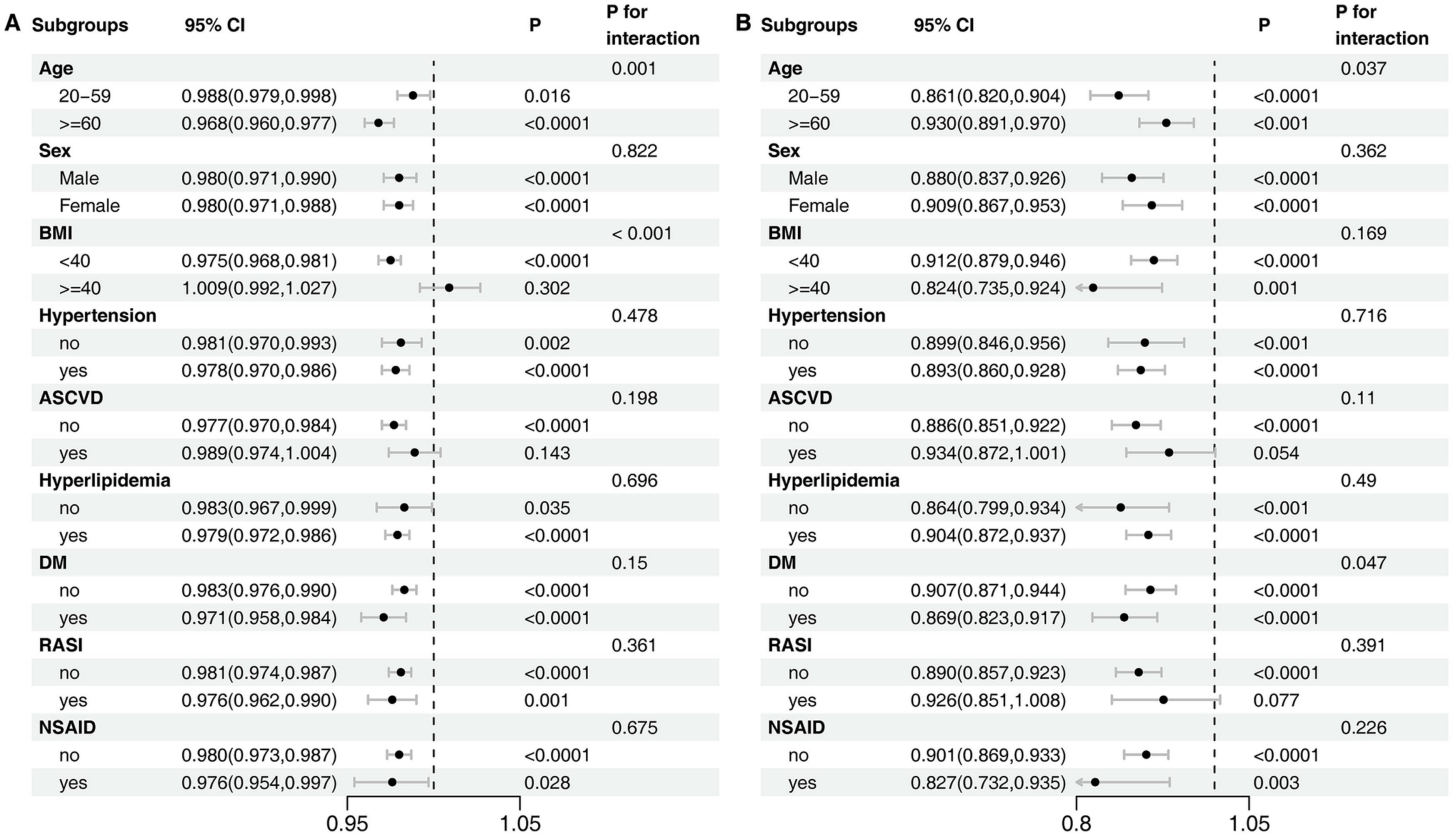
Subgroup Analyses of the Association between OBS Components and CKD. **(A)** Dietary score. **(B)** Lifestyle score. The model was adjusted for age, sex, race/ethnicity, marital status, education, hypertension, hyperlipidemia, diabetes mellitus, ASCVD, RASI, and NSAID.

A second sensitivity analysis was performed to explore the independent contributions of the OBS dietary and lifestyle components. In fully adjusted models, both the dietary score (OR, 0.979; 95% CI, 0.972–0.985) and the lifestyle score (OR, 0.874; 95% CI, 0.844–0.904) were independently associated with lower odds of CKD (Supplementary Tables S4–S7). Consistent with the total OBS analysis, restricted cubic spline models for both the dietary score (*P*_non-linearity_ = 0.052) and the lifestyle score (*P*_non-linearity_ = 0.061) indicated predominantly linear inverse associations with CKD risk (Supplementary Figures S3, S4). Subgroup analyses of these components provided further insight, particularly regarding the effect modification by morbid obesity. This analysis revealed that the protective effect of the dietary score was abrogated in individuals with a BMI ≥ 40 kg/m^2^ (*P*_interaction_ < 0.001) (Figure 4A). In contrast, the lifestyle score remained significantly protective in this same subgroup, with no evidence that its effect was modified by morbid obesity (*P*_interaction_ = 0.169) (Figure 4B).

### Comparison of machine learning model performance for CKD prediction

**Figure 5:**
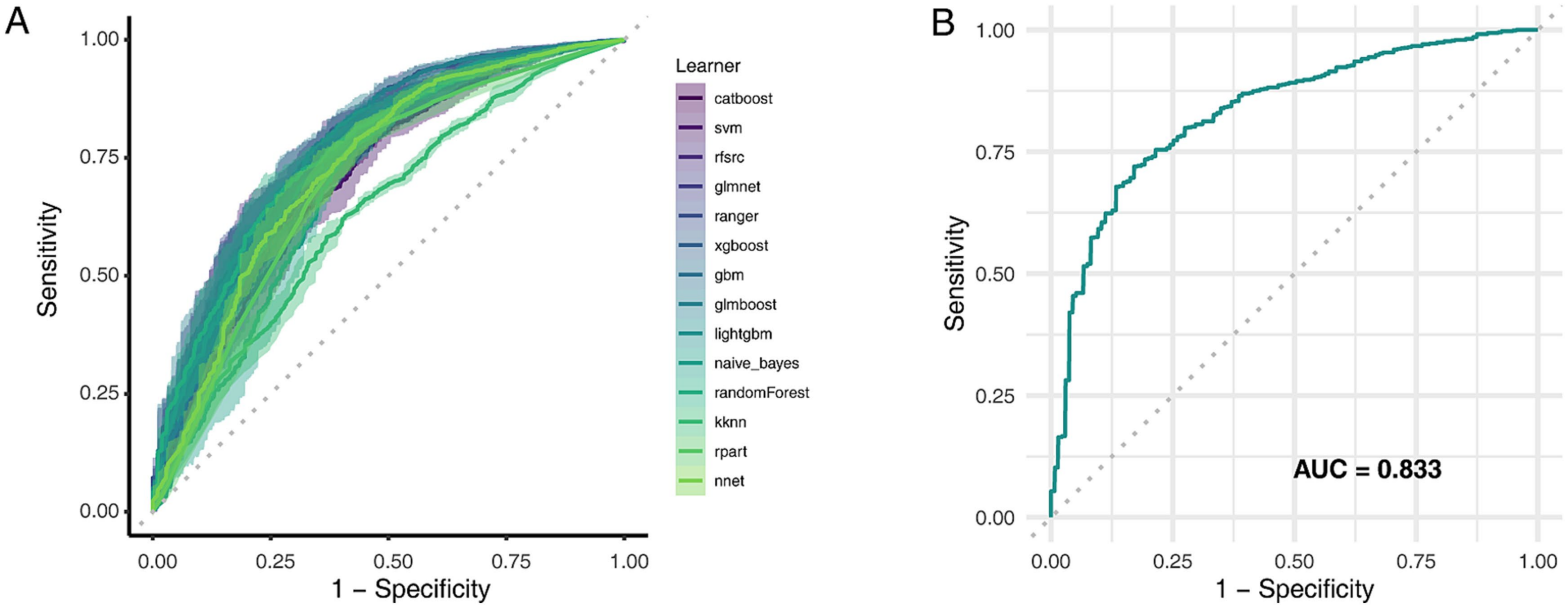
Comparative performance of machine learning models in predicting CKD risk. **(A)** AUC values for 14 classification algorithms during five-fold cross-validation. **(B)** ROC curves of selected models evaluated on the test set. AUC, Area under the curve; ROC, Receiver operating characteristic.

Fourteen machine learning algorithms were evaluated via five-fold cross-validation on the training data to identify the optimal model. The GLMBoost algorithm achieved the highest mean area under the receiver operating characteristic curve (AUC) during cross-validation (0.778; Figure 5A; Supplementary Table S8) and was therefore selected for final evaluation. When applied to the independent internal test set, the final GLMBoost model demonstrated strong predictive performance with an AUC of 0.833 (Figure 5B), an accuracy of 77.0%, a precision of 94.9%, a recall of 77.3%, and an F1-score of 85.2%. These findings confirm the robustness and generalizability of the selected model for CKD risk prediction in this population.

### SHAP-based interpretation and feature importance

**Figure 6:**
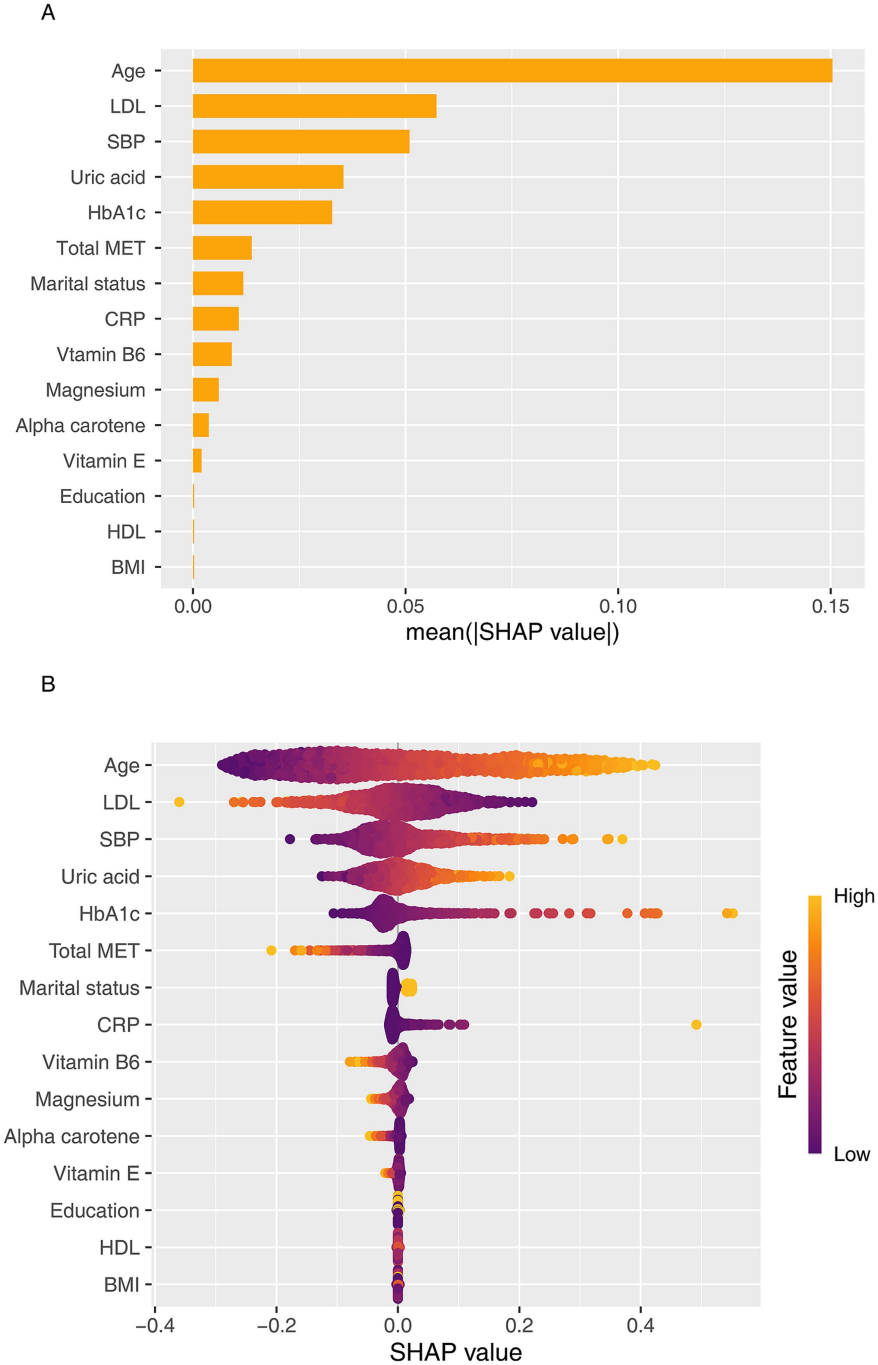
SHAP-based interpretation of the GLMBoost model for predicting CKD risk. **(A)** SHAP summary plot showing the global importance and directionality of top features contributing to CKD prediction. **(B)** SHAP beeswarm plot visualizing individualized feature contributions across participants. LDL-C, Low-density lipoprotein cholesterol; SBP, Systolic blood pressure; HbA1c, Hemoglobin A1c; CRP, C-reactive protein.

Figure 6 illustrates the SHAP-based interpretability analysis of the GLMBoost model, encompassing both global feature importance (Figure 6A) and individualized feature contributions (Figure 6B). The SHAP framework quantifies the marginal impact of each predictor on the model’s output, enabling both global and local interpretation of CKD risk predictions. At the global level, age emerged as the most influential predictor, as indicated by the highest mean SHAP value, highlighting its central role in CKD pathophysiology. This was followed by LDL-C and SBP, underscoring the contribution of cardiometabolic risk factors to renal function decline. Other top-ranking features included serum uric acid, HbA1c, total MET, marital status, CRP, and dietary intakes of vitamin B6 and magnesium. Notably, multiple micronutrients included in the OBS were consistently ranked among the most impactful features, reinforcing the significance of nutritional exposures in CKD risk stratification. The SHAP beeswarm plot (Figure 6B) further visualized the directionality of each feature’s effect. Higher values of age and LDL-C were associated with positive SHAP values, indicating increased CKD risk, whereas elevated levels of physical activity, magnesium, and vitamin B6 were associated with negative SHAP values, suggesting potential protective effects.

**Figure 7:**
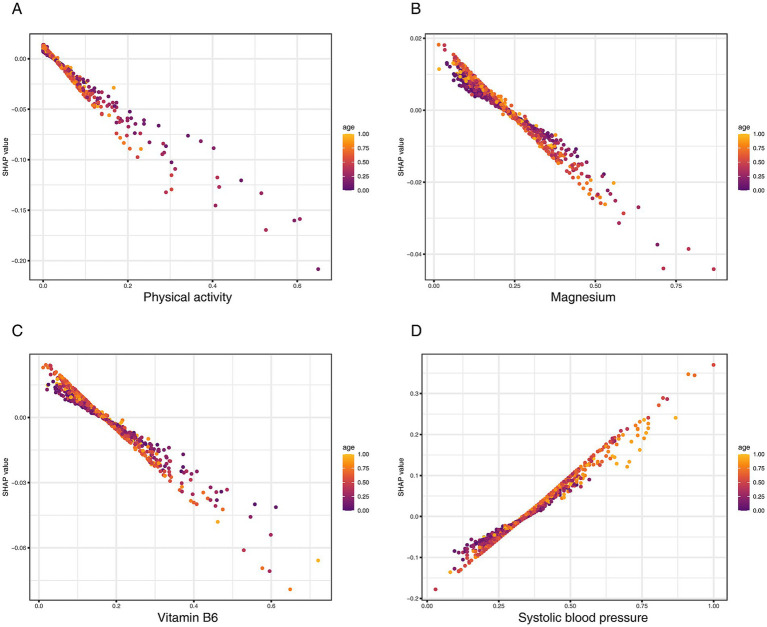
SHAP dependence plots visualizing the interaction of key predictors with age on CKD risk. **(A)** Physical activity (total MET), **(B)** magnesium, **(C)** vitamin B6, and **(D)** systolic blood pressure.

To further explore the interplay among top predictors, we generated SHAP dependence plots, focusing on age as a potential effect modifier (Figure 7). These plots revealed that the protective effects of key OBS components, including physical activity (Figure 7A), Magnesium (Figure 7B), and vitamin B6 (Figure 7C), were more pronounced in older individuals. Conversely, the analysis showed a strong synergistic interaction between age and systolic blood pressure, where the risk conferred by hypertension was substantially amplified in the elderly population (Figure 7D). Taken together, these analyses reveal that age critically modulates the impact of both protective lifestyle factors and traditional clinical risk factors on CKD risk.

## Discussion

This study, leveraging nationally representative data from NHANES, is the first to systematically examine the association between OBS and CKD specifically among overweight and obese adults. By integrating traditional survey-weighted logistic regression with SHAP-enhanced machine learning techniques, we evaluated the predictive value of OBS for CKD from both statistical and interpretative standpoints. The incorporation of SHAP analysis enhanced model transparency and allowed for the quantification of individual feature contributions, providing novel insights into the role of specific OBS components in CKD risk stratification.

Our study’s primary finding—a significant inverse association between OBS and CKD—builds upon the established role of oxidative stress as a key pathological driver in obesity-related diseases. The OBS is an integrative metric designed to provide a holistic estimate of an individual’s systemic oxidative state by synthesizing numerous dietary and lifestyle factors into a single, quantifiable score. Its utility has been previously demonstrated through associations with various cardiometabolic outcomes, including hypertension, type 2 diabetes, and mortality (14–18). However, its specific application to CKD risk within the large and growing population of overweight and obese adults has remained underexplored (19). This study addresses that gap, providing evidence for the utility of this composite lifestyle score in a high-risk clinical context where metabolic dysfunction and inflammation are prevalent (20). Our study confirms a significant and linear inverse association between OBS and CKD risk specifically within this large cohort, which persisted after comprehensive adjustment for demographic, clinical, and medication-related confounders. This dose–response relationship suggests that any incremental improvement in oxidative balance may confer a protective benefit.

Our subgroup analyses revealed a critical interaction by obesity status (*P*_interaction_ < 0.001). While a higher OBS was consistently protective in individuals with a BMI < 40 kg/m^2^, this overall association was attenuated to the null in the morbidly obese group. To explore this further, our sensitivity analysis deconstructed the OBS into its lifestyle and dietary components, which provided a crucial insight. This analysis revealed that the loss of association was driven entirely by the dietary score, which showed no significant protective effect in the morbidly obese subgroup. In striking contrast, a higher lifestyle score, which includes physical activity, remained significantly associated with lower odds of CKD even in individuals with a BMI ≥ 40 kg/m^2^.

This suggests a potential “threshold effect” of severe obesity, where the overwhelming systemic inflammation and metabolic dysregulation may blunt the benefits of dietary antioxidants (21, 22). Pathological processes such as irreversible podocyte loss and advanced glomerulosclerosis may reach a point where the benefits of an improved dietary oxidative balance are marginal (23). However, the protective mechanisms associated with key lifestyle behaviors—such as improved insulin sensitivity and endothelial function from physical activity—appear to be more robust and remain impactful (24, 25).

The application of SHAP analysis provided a granular view of the predictors driving CKD risk in our model. While confirming the dominant role of traditional risk factors like age and systolic blood pressure, the analysis also highlighted several modifiable OBS components as significant contributors. Physical activity (Total MET) emerged as the most important protective lifestyle factor, reinforcing the independent renoprotective role of exercise through its known benefits on insulin sensitivity and systemic inflammation (26). Among nutritional factors, higher intakes of vitamin B6, magnesium, and antioxidant carotenoids like α-carotene were also associated with lower CKD risk. These components likely exert their protective effects through complementary mechanisms, including the facilitation of homocysteine metabolism (vitamin B6) (27) and the support of renal vascular integrity (magnesium) (28). It is important to contextualize these findings within the broader evidence base; while our results underscore the importance of dietary patterns rich in these nutrients, high-dose single-nutrient supplementation has shown inconsistent results for renal outcomes in clinical trials (29–31).

Beyond ranking individual features, our interaction analysis revealed a critical dynamic: the pervasive influence of age as an effect modifier. We observed a strong synergistic effect where the risk conferred by hypertension was substantially amplified in older individuals, suggesting a heightened vulnerability of the aging kidney to hemodynamic stress (32–34). Conversely, the protective effects of key OBS components, including physical activity and vitamin B6, were also more pronounced in the elderly. This novel finding may indicate that older adults, who are more likely to have a higher baseline of oxidative stress, derive a greater relative benefit from positive antioxidant and anti-inflammatory lifestyle inputs (35). These interactions highlight the importance of viewing CKD risk not as a set of static factors, but as a dynamic interplay where the impact of modifiable behaviors is critically modulated by the aging process (36, 37).

As a composite measure of modifiable behaviors, the OBS can serve as a practical, non-invasive tool for primary prevention, helping clinicians identify at-risk overweight individuals before clinical markers of kidney damage appear (38). It does not replace traditional risk factors but rather complements them by providing a quantifiable summary of a patient’s lifestyle-related risk profile (39). Our results suggest that intervention strategies based on the OBS could be tailored by obesity severity. For the majority of overweight and obese individuals (BMI < 40 kg/m^2^), a low OBS score can trigger targeted counseling focused on improving specific components identified by our SHAP analysis. This includes increasing physical activity and ensuring adequate dietary intake of foods rich in vitamin B6 and magnesium. However, for individuals with morbid obesity (BMI ≥ 40 kg/m^2^), our findings suggest that while improving the lifestyle components of OBS is still crucial, the benefits of dietary improvements may be diminished. This underscores the need for more intensive primary interventions for this group, such as structured weight management programs, in addition to behavioral counseling (21, 40).

Our study has several notable strengths, including its large, nationally representative cohort, comprehensive adjustment for confounders, and the innovative application of interpretable machine learning. Nevertheless, certain limitations warrant consideration. First, the OBS was calculated from 24-h dietary recalls, which are subject to recall bias and may not reflect long-term habitual intake, a common consideration in large-scale nutritional epidemiology. Second, the study’s cross-sectional design establishes association but does not permit causal inference; future prospective cohort studies are warranted to confirm the temporal relationship between OBS and the incidence of CKD. Third, our analysis relied on the OBS as a proxy for oxidative stress, as direct measurement of circulating antioxidant levels or renal-specific biomarkers was not available within the NHANES dataset. Integrating such measures in future studies could provide deeper mechanistic insights. Fourth, our findings are based on a representative sample of the U. S. population and their generalizability to populations with different ethnic or dietary backgrounds requires further investigation. Finally, while our machine learning models demonstrated strong performance with internal validation, their transportability to other settings and their real-world clinical utility must be established through external validation in independent, prospective cohorts. This represents a crucial next step for translating these predictive models into clinical practice.

## Conclusion

This study, based on a large and nationally representative cohort of overweight and obese U. S. adults, demonstrates that a higher OBS is consistently associated with lower odds of CKD. Importantly, this protective association appears to be primarily driven by the lifestyle components of the OBS, which retained their effect even among individuals with morbid obesity—a condition in which the benefits of dietary components were markedly diminished. Additionally, our findings highlight age as a key effect modifier, amplifying both the adverse impact of clinical risk factors and the protective influence of health-promoting behaviors. Together, these results position the OBS as a valuable integrative framework for capturing the complex interplay between modifiable risk factors and renal health. Although these associations warrant validation in prospective studies, the OBS holds significant potential as a practical tool for guiding personalized, preventative strategies in high-risk populations with obesity.

## Data Availability

Publicly available datasets were analyzed in this study. This data can be found here: The data derived from the National Health and Nutrition Examination Survey can be publicly accessed at https://wwwn.cde.gov/nchs/nhanes.
