# Supplementary material for: Oxidative balance score predicts chronic kidney disease risk in overweight adults: a NHANES-based machine learning study
Source: Front Nutr. 2025 Jul 16;12:1641496. doi: 10.3389/fnut.2025.1641496 (PMC12307168; doi:10.3389/fnut.2025.1641496)
Supplement: Supplementary file 1 [file Data_Sheet_1.PDF]

Supplementary Table S1: Components of the Oxidative Balance Score (OBS)

Component	Classification
Dietary Antioxidants	
Dietary Fiber	Antioxidant
Carotenoids	Antioxidant
Riboflavin (Vitamin B2)	Antioxidant
Niacin (Vitamin B3)	Antioxidant
Vitamin B6	Antioxidant
Vitamin B12	Antioxidant
Vitamin C	Antioxidant
Vitamin E	Antioxidant
Total Folate	Antioxidant
Calcium	Antioxidant
Magnesium	Antioxidant
Zinc	Antioxidant
Copper	Antioxidant
Selenium	Antioxidant
Lifestyle Antioxidant	
Physical Activity	Antioxidant
Dietary Pro-oxidants	
Total Fat	Pro-oxidant
Iron	Pro-oxidant
Lifestyle Pro-oxidants	
Alcohol Intake	Pro-oxidant
Serum Cotinine (Smoking Exposure)	Pro-oxidant
Body Mass Index (BMI)	Pro-oxidant

Supplementary Table S2: Association between Continuous Oxidative Balance Score and CKD Risk in the Sensitivity Analysis Cohort (Excluding Participants with High- and Very-High-Risk CKD).

	OBS	P
crude model	0.973(0.966,0.979)	<0.0001
Model 1	0.977(0.970,0.984)	<0.0001
Model 2	0.980(0.973,0.987)	<0.0001
Model 3	0.980(0.972,0.987)	<0.0001

model 1: ethnicity, age, sex, education, marital status

model 2: ethnicity, age, sex, education, marital status, hypertension, hyperlipidemia, ASCVD, DM

model 3: ethnicity, age, sex, education, marital status, hypertension, hyperlipidemia, ASCVD, DM, RASI, NSAID

Supplementary Table S3: Association between Quartiles of Oxidative Balance Score and CKD Risk in the Sensitivity Analysis Cohort (Excluding Participants with High- and Very-High-Risk CKD).

	Q1	Q2	*P*	Q3	*P*	Q4	*P*	*P* for trend
crude model	ref	0.843(0.742,0.957)	0.009	0.694(0.616,0.782)	<0.0001	0.607(0.528,0.698)	<0.0001	<0.0001
Model 1	ref	0.841(0.737,0.961)	0.011	0.725(0.641,0.819)	<0.0001	0.659(0.569,0.763)	<0.0001	<0.0001
Model 2	ref	0.844(0.733,0.971)	0.018	0.744(0.654,0.845)	<0.0001	0.693(0.596,0.806)	<0.0001	<0.0001
Model 3	ref	0.839(0.728,0.965)	0.015	0.740(0.650,0.842)	<0.0001	0.689(0.592,0.802)	<0.0001	<0.0001

Low exposure (Q1) was used as the reference group.

model 1: ethnicity, age, sex, education, marital status

model 2: ethnicity, age, sex, education, marital status, hypertension, hyperlipidemia, ASCVD, DM

model 3: ethnicity, age, sex, education, marital status, hypertension, hyperlipidemia, ASCVD, DM, RASI, NSAID

Supplementary Table S4: Association between the Continuous Dietary Score and CKD Risk in the Fully Adjusted Model.

	OBS.dietary	*P*
crude model	0.966(0.961,0.972)	<0.0001
Model 1	0.976(0.970,0.982)	<0.0001
Model 2	0.979(0.973,0.985)	<0.0001
Model 3	0.979(0.972,0.985)	<0.0001

model 1: ethnicity, age, sex, education, marital status

model 2: ethnicity, age, sex, education, marital status, hypertension, hyperlipidemia, ASCVD, DM

model 3: ethnicity, age, sex, education, marital status, hypertension, hyperlipidemia, ASCVD, DM, RASI, NSAID

Supplementary Table S5: Association between Quartiles of the Dietary Score and CKD Risk in the Fully Adjusted Model.

	Q1	Q2	*P*	Q3	*P*	Q4	*P*	*P* for trend
crude model	ref	0.761(0.690,0.839)	<0.0001	0.658(0.598,0.724)	<0.0001	0.558(0.498,0.626)	<0.0001	<0.0001
Model 1	ref	0.777(0.695,0.869)	<0.0001	0.728(0.655,0.809)	<0.0001	0.674(0.596,0.763)	<0.0001	<0.0001
Model 2	ref	0.778(0.693,0.874)	<0.0001	0.753(0.674,0.842)	<0.0001	0.706(0.620,0.805)	<0.0001	<0.0001
Model 3	ref	0.773(0.687,0.868)	<0.0001	0.749(0.669,0.838)	<0.0001	0.701(0.615,0.798)	<0.0001	<0.0001

Low exposure (Q1) was used as the reference group.

model 1: ethnicity, age, sex, education, marital status

model 2: ethnicity, age, sex, education, marital status, hypertension, hyperlipidemia, ASCVD, DM

model 3: ethnicity, age, sex, education, marital status, hypertension, hyperlipidemia, ASCVD, DM, RASI, NSAID

Supplementary Table S6: Association between the Continuous Lifestyle Score and CKD Risk in the Fully Adjusted Model.

	OBS.lifestyle	P
crude model	0.875(0.848,0.903)	<0.0001
Model 1	0.839(0.811,0.868)	<0.0001
Model 2	0.877(0.847,0.907)	<0.0001
Model 3	0.874(0.844,0.904)	<0.0001

model 1: ethnicity, age, sex, education, marital status

model 2: ethnicity, age, sex, education, marital status, hypertension, hyperlipidemia, ASCVD, DM

model 3: ethnicity, age, sex, education, marital status, hypertension, hyperlipidemia, ASCVD, DM, RASI, NSAID

Supplementary Table S7: Association between Quartiles of the Lifestyle Score and CKD Risk in the Fully Adjusted Model.

	Q1	Q2	*P*	Q3	*P*	Q4	*P*	*P* for trend
crude model	ref	0.951(0.859,1.053)	0.329	0.747(0.657,0.851)	<0.0001	0.580(0.509,0.661)	<0.0001	<0.0001
Model 1	ref	0.846(0.755,0.947)	0.004	0.629(0.549,0.721)	<0.0001	0.508(0.442,0.583)	<0.0001	<0.0001
Model 2	ref	0.873(0.779,0.978)	0.020	0.708(0.619,0.810)	<0.0001	0.590(0.514,0.677)	<0.0001	<0.0001
Model 3	ref	0.865(0.771,0.971)	0.014	0.700(0.612,0.802)	<0.0001	0.584(0.508,0.670)	<0.0001	<0.0001

Low exposure (Q1) was used as the reference group.

model 1: ethnicity, age, sex, education, marital status

model 2: ethnicity, age, sex, education, marital status, hypertension, hyperlipidemia, ASCVD, DM

model 3: ethnicity, age, sex, education, marital status, hypertension, hyperlipidemia, ASCVD, DM, RASI, NSAID

Supplementary Table S8. Predictive performance of fourteen machine learning models for chronic kidney disease risk classification in overweight adults

learner_id	Resampling method	auc	acc	recall	precision	fbeta
catboost	5-fold cross-validation	0.746071	0.854605	0.932344	0.903549	0.917656
svm	5-fold cross-validation	0.708357	0.803089	0.863735	0.905457	0.884034
rfsrc	5-fold cross-validation	0.76644	0.838917	0.903139	0.910948	0.906908
glmnet	5-fold cross-validation	0.776628	0.744512	0.756325	0.937777	0.837227
ranger	5-fold cross-validation	0.759093	0.845976	0.924207	0.901222	0.912534
xgboost	5-fold cross-validation	0.731437	0.855131	0.934721	0.90217	0.918107
gbm	5-fold cross-validation	0.749063	0.770401	0.799375	0.926619	0.858187
glmboost	5-fold cross-validation	0.778165	0.741898	0.752421	0.938563	0.835092
lightgbm	5-fold cross-validation	0.751151	0.85905	0.932622	0.907814	0.919991
naive_bayes	5-fold cross-validation	0.698207	0.575824	0.555554	0.927817	0.693446
randomForest	5-fold cross-validation	0.760965	0.84467	0.920285	0.902971	0.911488
kknn	5-fold cross-validation	0.635894	0.688023	0.723275	0.898062	0.80117
rpart	5-fold cross-validation	0.701432	0.748699	0.775192	0.924053	0.842307
nnet	5-fold cross-validation	0.730584	0.749213	0.772261	0.927786	0.841131

Supplementary Figure S1: Restricted Cubic Spline for the Association between Oxidative Balance Score and CKD Risk in the Sensitivity Analysis Cohort.

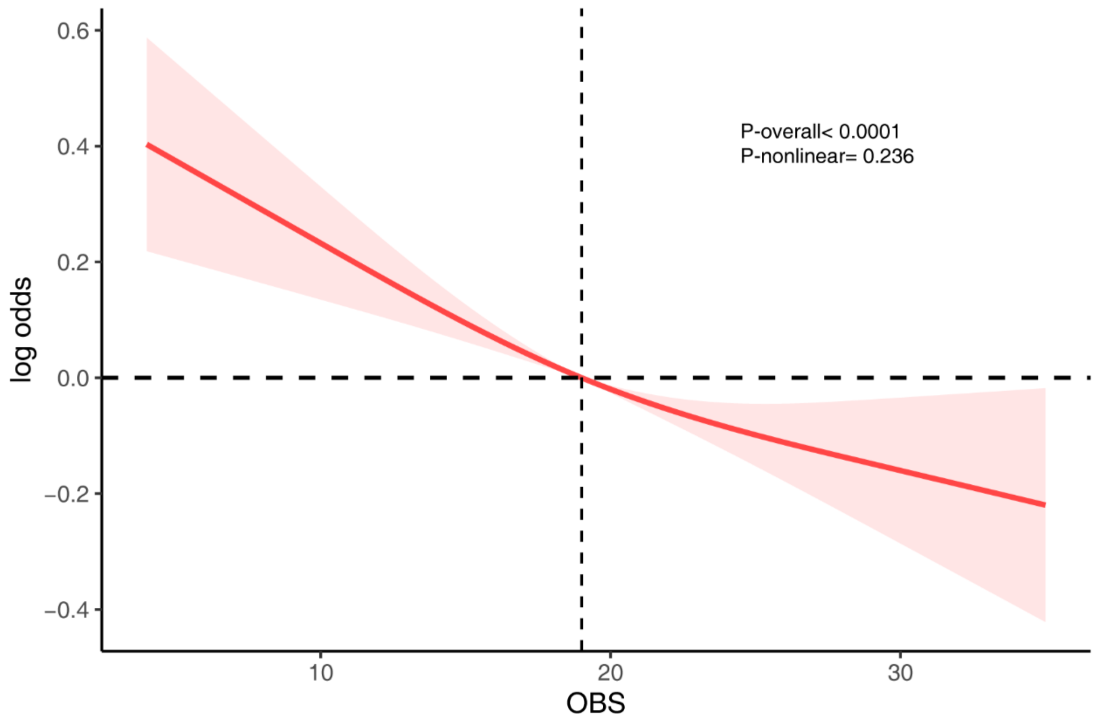

The model was adjusted for age, sex, race/ethnicity, marital status, education, hypertension, hyperlipidemia, diabetes mellitus, ASCVD, RASI and NSAID.

Supplementary Figure S2: Subgroup Analysis of the Association between Oxidative Balance Score and CKD Risk in the Sensitivity Analysis Cohort.

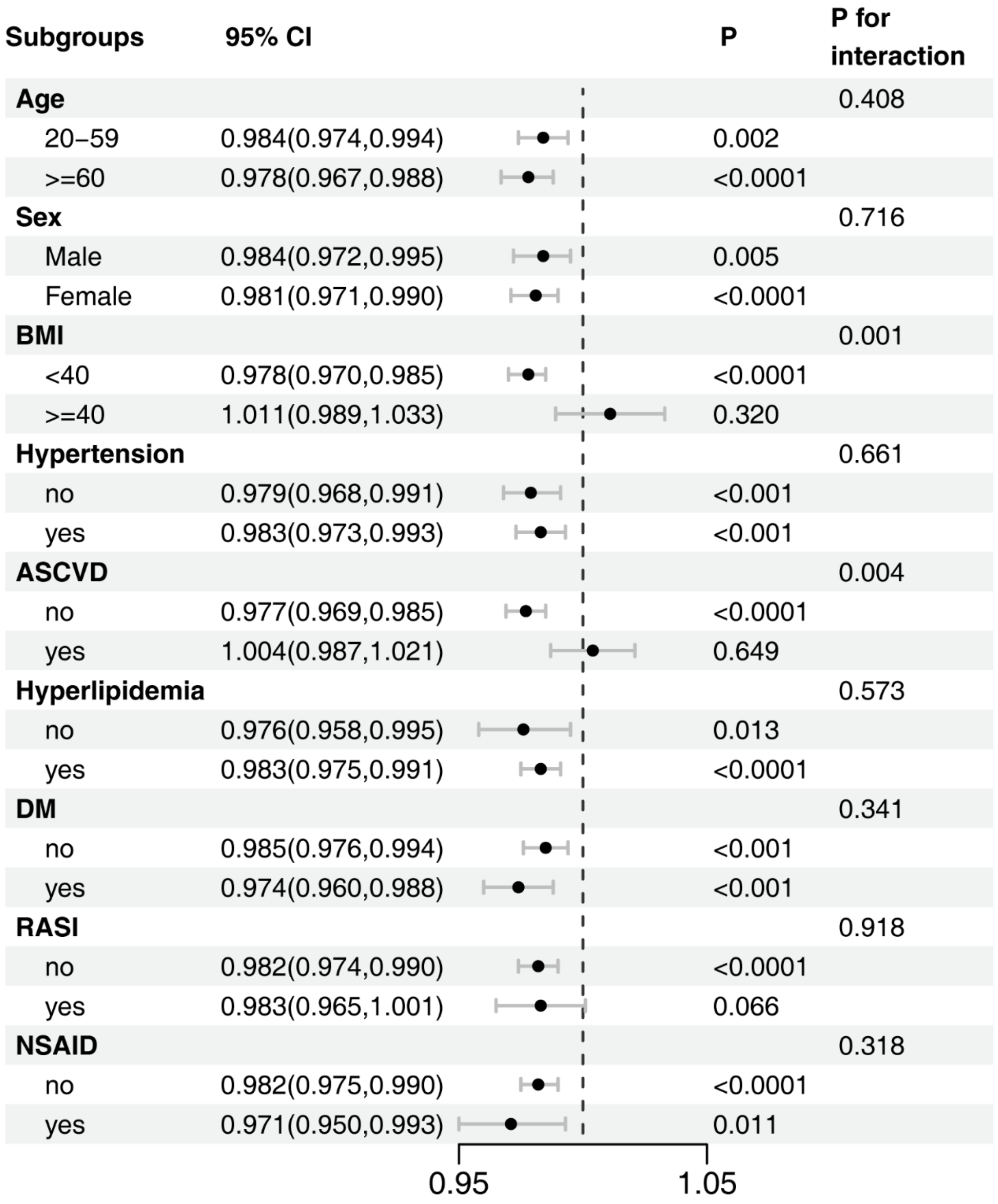

The model was adjusted for age, sex, race/ethnicity, marital status, education, hypertension, hyperlipidemia, diabetes mellitus, ASCVD, RASI and NSAID.

Supplementary Figure S3: Restricted Cubic Spline for the Association between the Dietary Score and CKD Risk.

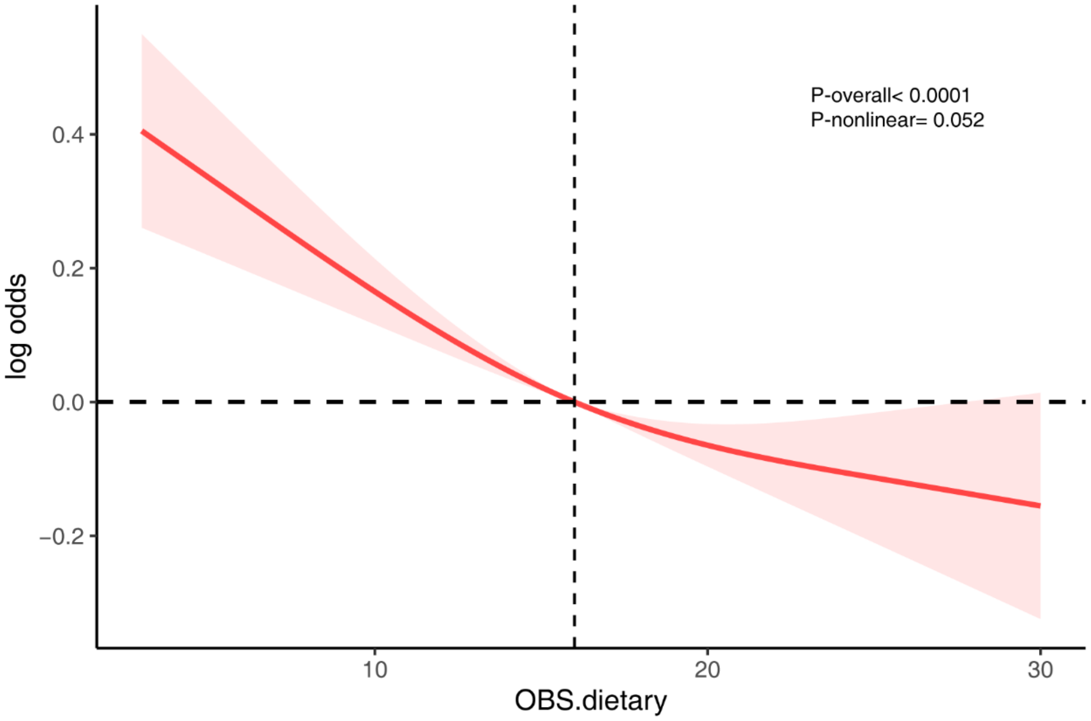

The model was adjusted for age, sex, race/ethnicity, marital status, education, hypertension, hyperlipidemia, diabetes mellitus, ASCVD, RASI and NSAID.

Supplementary Figure S4: Restricted Cubic Spline for the Association between the Lifestyle Score and CKD Risk.

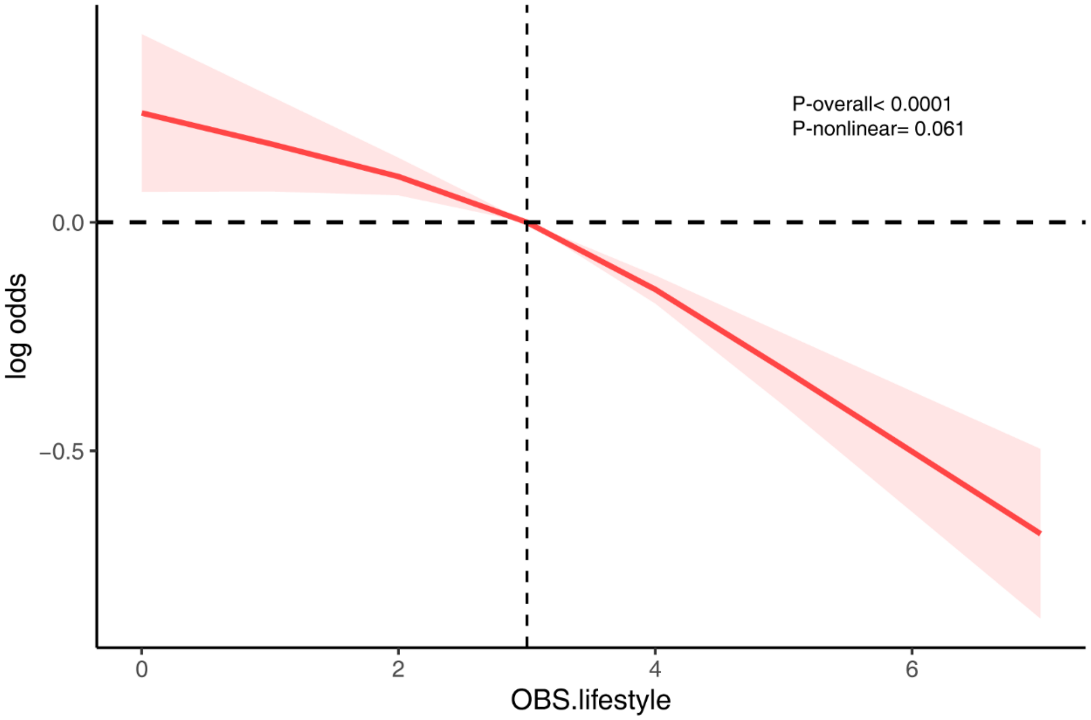

The model was adjusted for age, sex, race/ethnicity, marital status, education, hypertension, hyperlipidemia, diabetes mellitus, ASCVD, RASI and NSAID.
